# Heterotopic ossification following hip arthroplasty: a comparative radiographic study about its development with the use of three different kinds of implants

**DOI:** 10.1186/s13018-015-0317-2

**Published:** 2015-11-14

**Authors:** Carlo Biz, Davide Pavan, Antonio Frizziero, Ala Baban, Claudio Iacobellis

**Affiliations:** Orthopaedic and Traumatology Clinic, Department of Surgery, Oncology and Gastroenterology DiSCOG, University of Padua, via Giustiniani 2, 35128 Padova, Italy; Department of Orthopaedic Rehabilitation, University of Padua, via Giustiniani 2, 35128 Padova, Italy

**Keywords:** Heterotopic ossification, Periarticular ossification, Hip arthroplasty, Hip endoprosthesis, Prophylactic therapy

## Abstract

**Background:**

Our purpose was to record the incidence of heterotopic ossification (HO) following hip replacement by different variables to identify patient groups that are likely to develop HO in the absence of a prophylactic protocol.

**Methods:**

Radiographically, we studied 651 patients having undergone hip joint replacement, evaluating three kinds of implants: *ceramic-ceramic-coupled total hip replacement* (THR), *TriboFit*^*®*^ with polycarbonate urethane-ceramic coupling and *endoprosthesis*. Each patient was analysed for HO development by age, gender, diagnosis, presence of previous ossifications, surgical approach and kind of implant. Within the population that developed HO, data were assessed for correlation with severity of ossification graded according to Brooker classification.

**Results:**

The overall incidence of HOs was 59.91 %. The factors increasing their incidence in the univariate analysis were as follows: lower age of the patients with HO (mean 77.6 years, *p* = 0.0018) than those subjects who did not develop HO (mean 80.2 years); male gender (64.4 %, *p* = 0.1011); diagnosis of coxarthrosis (72.7 %, *p* = 0.0001) compared to femur neck fracture (55.9 %, *p* = 0.0001); presence of previous HO (76.2 %, *p* = 0.0260); lateral approach (65.5 %) as opposed to anterior-lateral approach (55.6 %, *p* = 0.0163); and ceramic-ceramic THR (68.1 %) and TriboFit^®^ (67.0 %) compared to endoprosthesis (51.3 %, *p* = 0.0001).

During multivariate analysis, the presence of HO after previous hip surgery (*p* = 0.0324) and the kind of implant (*p* = 0.0004) showed to be independent risk factors for the development of HO. Analysing the population that developed HO, we found that the severity of ossification by Brooker classification was influenced by gender (*p* = 0.0478) and kind of implant (*p* = 0.0093).

**Conclusions:**

In agreement with the literature, our radiographic study confirms the following risk factors of HO development in absence of any prophylactic treatment: male gender, diagnosis of coxarthrosis compared to femur neck fracture, previous HO, surgical approach and kind of implant. In particular, Hardinge-Bauer and Watson-Jones surgical approaches, characterized by a wide exposure of the coxofemoral joint, and ceramic-ceramic THR and TriboFit^®^ implants significantly increase the development of HO.

## Background

Heterotopic ossification (HO) is the presence of the lamellar bone within soft tissues where the bone physiologically does not exist. One of the most common forms of HO is that which intervenes in periarticular soft tissue after hip replacement, with a mean incidence of 53 % reported in the literature [1]. Multiple studies have been performed to date, but aetiopathogenesis of HO is still uncertain. Several authors [2–11] have confirmed some risk factors including male gender, presence of prior HO, previous hip surgery and lateral and anterolateral hip approaches. Some pathologies have been associated with a higher rate of HO, such as ankylosing spondylitis, hypertrophic osteoarthritis, diffuse idiopathic skeletal hyperostosis, Paget disease, Parkinson’s disease and rheumatoid arthritis. The incidence of HO in hip surgery can be vastly different in different ethnic groups. Some studies [2–4, 12] reported a higher incidence of HO in African-American patients following acetabular fracture surgery and in Japanese population because of spastic limb, traumatic brain injuries, spinal cord lesions, nerve injuries and neurological disorders, when compared with Europeans. The most widely used classification system for HO following hip arthroplasty was developed by Brooker et al. [13] in 1973. Effective measures (NSAIDs, radiation therapy and selective inhibitors of COX2) to prevent HO after prosthetic hip surgery are well documented [14–20]. However, there is no universal agreement as to which therapeutic protocol is the best.

The aim of this radiographic and retrospective study, performed on a wide cross-section of patients having undergone hip replacement, was to record the incidence of HO by the following variables: three different kinds of implants, surgical approach, pre-existing HO, age, sex and diagnosis of diseased hip, as well as identify patient groups that are likely to develop HO in the absence of a prophylactic protocol.

## Materials and methods

Collection and statistical analysis of data were performed at our Orthopaedic and Traumatology Clinic during a period of 24 months, from September 2012 to August 2014, by an external and independent investigator (PD) not involved in the patients’ treatment. Information matter of the research was learnt consulting retrospective case histories, surgical procedures and radiographic reports from the computerized archives of our hospital. Radiographic data of HO were obtained reading the radiographic computerized images taken in the preoperative, postoperative and follow-up periods, available in the computer system of our institute. We used a diagnostic LCD CORONIS 3MP display (produced by Barco, Rome, Italy) as a viewing monitor to determine the presence and extent of HO.

To classify HOs, we used Brooker classification that identifies four grades of HO based on an anteroposterior radiograph of the pelvis:

- Grade 1: the presence of isolated bone fragments of any size within periarticular soft tissue
- Grade 2: the presence of bone spurs from the pelvis or femur with at least 1 cm between opposing bone surfaces
- Grade 3: the presence of bone spurs reducing space between opposing bone surfaces to less than 1 cm
- Grade 4: ossification with apparent ankylosis of the hip

In this study, we examined data from a total of 823 patients who underwent, from 2006 to 2013, a surgical hip replacement in our Orthopaedic Clinic with one of the following kinds of implant: *ceramic-ceramic total hip replacement* (THR), the *TriboFit*^*®*^*system* with polycarbonate urethane-ceramic coupling and *endoprosthesis*. All subjects participating in this study received a thorough explanation of this study and gave their oral and written informed consent to publish the data. The study was performed in accordance with the ethical standards of the 1964 Declaration of Helsinki as revised in 2000.

Patients were included in the study on the basis of the presence in our computerized database of a minimum follow-up control at 6 months from the surgical intervention (mean follow-up time 32.66 ± 13.17 months; range 6–72 months). This period was considered the sufficient minimum to observe the development of HO after hip surgery. With this premise, data from a total of 651 patients were analysed. None of the patients studied was treated with prophylactic drugs against formation of periarticular HO or other preventive therapies.

The following information was obtained for each patient from our database:

- Preoperative: age, sex, diagnosis of diseased hip, pre-existing HO of the hip following prior surgery, previous hip surgery on the same side
- Operative: surgical approach to the hip, kind of implant
- Postoperative: presence or absence of periarticular HO in the surgical hip classified according to Brooker system as mild (grade 1), moderate (grade 2), severe (grade 3) and very severe (grade 4)

The collected data were statistically assessed for correlation with the development of HO, independently from their grade, using the chi-square test of independence with a *p* value <0.05 as significance level. Then, factors found to significantly increase development of ectopic bone were compared individually using multivariate analysis (logistic regression) to rule out interdependence. Further, a possible relationship with statistical significance was analysed between each singular risk factor and the presence of HO considering its grade according to Brooker classification. Storage and statistical analysis of data were performed using SAS 9.2 (SAS Institute Inc., Cary, NC, USA) for Windows.

## Results

**Fig. 1:**
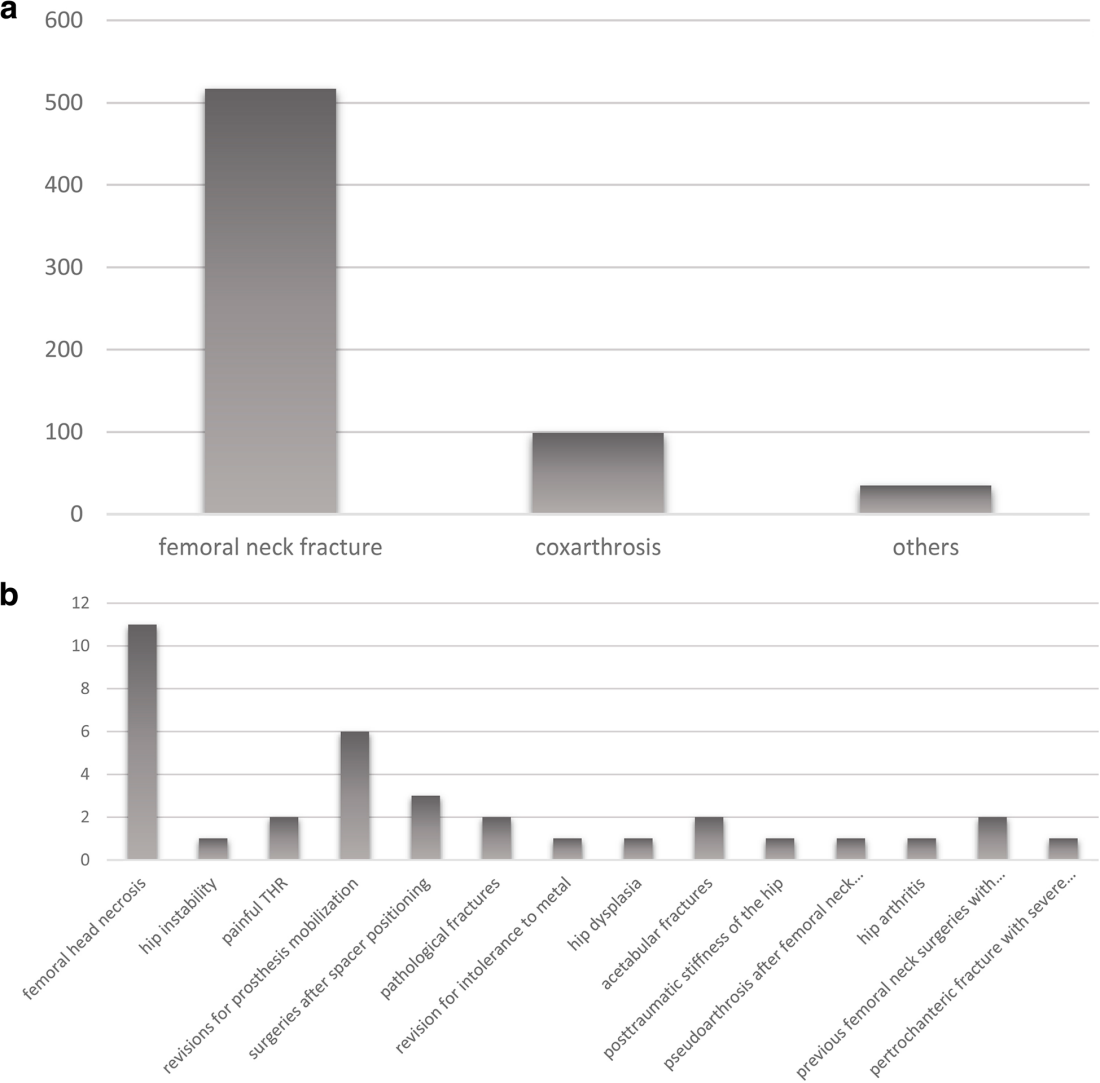
Distribution of the diagnoses of a diseased hip. **a** Main diagnoses. **b** Diagnoses included under “others”

The analysed cohort consisted of 440 women (67.59 %) and 211 men (32.41 %) with a mean patient age of 78.7 years (range 19–98 years) at the time of surgery. Diagnosis of diseased hip included traumatic femoral neck fracture in 517 patients (79.42 %), coxarthrosis in 99 patients (15.21 %) and other diagnoses in 35 patients (5.37 %). The latter included 11 femoral head necrosis, 1 hip instability, 2 painful THRs, 6 hip surgical revisions for prosthesis mobilization, 3 surgeries after spacer positioning, 2 pathological fractures, 1 revision for intolerance to metal, 1 hip dysplasia, 2 acetabular fractures, 1 posttraumatic stiffness of the hip, 1 pseudoarthrosis after femoral neck surgery with percutaneous screws, 1 hip arthritis, 2 previous femoral neck surgeries with percutaneous screws and 1 pertrochanteric fracture with severe coxarthrosis (Fig. 1). Forty-two patients (6.45 %) had already developed HO after previous surgery of the ipsilateral and/or contralateral hip. With regard to the population which underwent ceramic-ceramic THR, 20 (8.73 %) patients had already undergone a previous surgical intervention of the ipsilateral coxofemoral joint. All operations were carried out adopting one of the following two surgical approaches to the hip:

1. Transgluteal approach (Hardinge-Bauer) in 223 (34.25 %) patients
2. Anterolateral approach (Watson-Jones) in 401 (61.60 %) patients

For 27 (4.15 %) subjects included in the study, there were no data available in our archives concerning the surgical approach to the hip.

Patients underwent hip surgery with three kinds of implants:

1. Ceramic-ceramic THR: 229 (35.18 %) patients
2. TriboFit^®^ system with polycarbonate urethane-ceramic coupling: 112 (17.20 %) patients
3. Endoprosthesis: 310 (47.62 %) patients

**Fig. 2:**
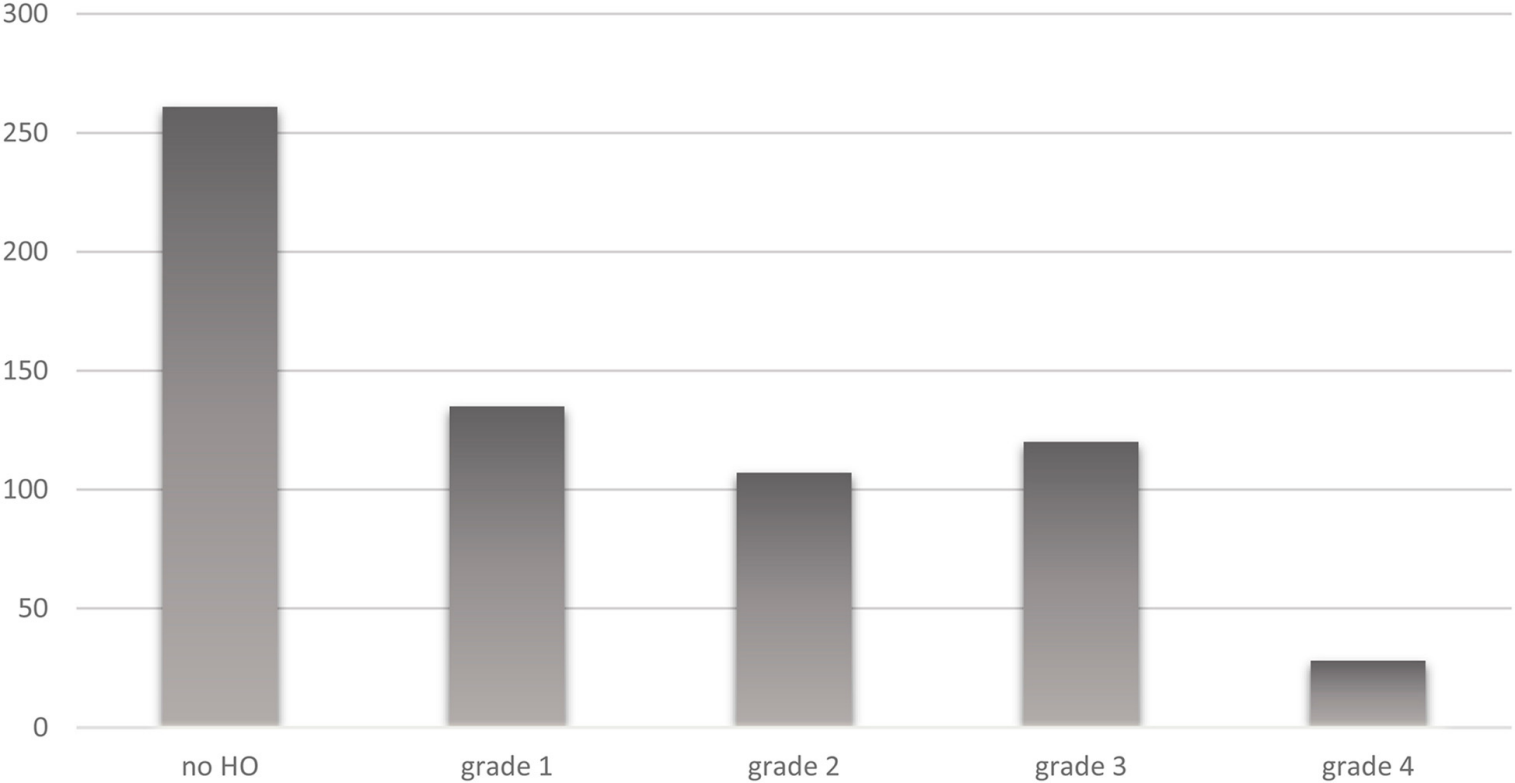
Frequencies of HO according to Brooker classification

**Fig. 3:**
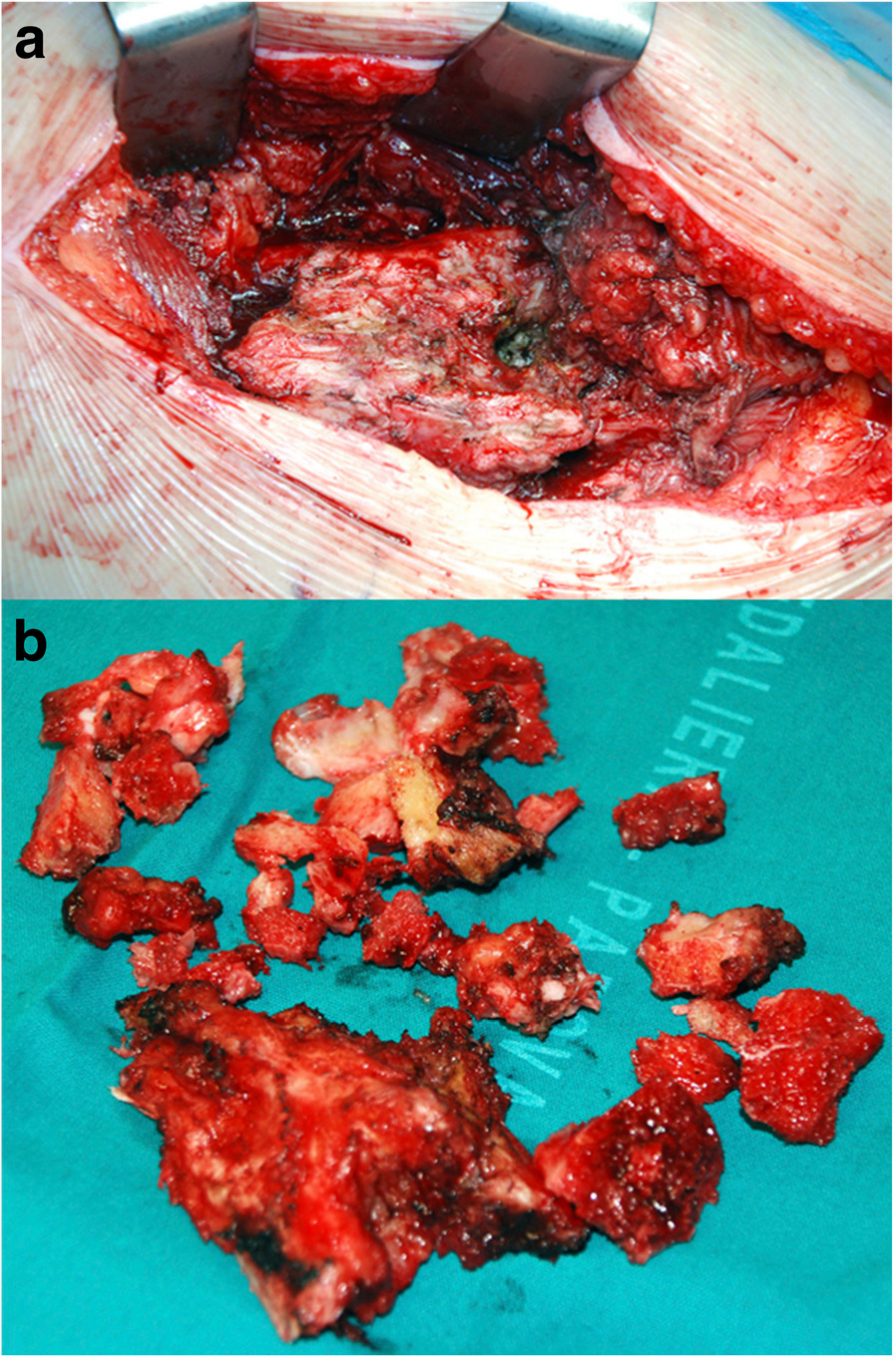
Case of severe heterotopic ossifications. HOs developed in a patient who had been moved to the emergency room after ceramic-ceramic THR because of her critical condition. The patient remained motionless for 1 month after hip replacement, and 1 year later, heterotopic ossifications were removed (**a**, **b**)

Periarticular HO formed in 59.9 % (390/651) of the patients (Fig. 2). Among these, 135 (34.6 %) had mild HO (grade 1, Brooker classification); 107 (27.4 %) had moderate HO (grade 2, Brooker classification); 120 (30.8 %) had severe HO (grade 3, Brooker classification); and 28 (7.2 %) showed very severe HO (grade 4, Brooker classification). The pictures taken at the operating table show grade 4 HOs that developed in a patient of our cohort who was moved to the emergency room because of her critical condition after the operation (Fig. 3).

Preoperative and operative independent variables were considered for those patients who developed periarticular HO after surgery as shown in Table 1. The mean patient age at the time of the surgery among those who showed HO formation was 77.6 years, compared to the mean age of 80.2 years among those who did not develop HO. Among male patients, 136 subjects formed HO, whereas among female they were 254. Periarticular ossification was found in 289 subjects with preoperative diagnosis of femoral neck fracture; 72 subjects with diagnosis of coxarthrosis and 29 subjects with other diagnoses. Among the population that had shown HO due to previous hip surgery, 32 patients developed HOs compared to 358 among those who had previously not had HO. Considering only the patients for whom ceramic-ceramic THR was used, HO developed in 15 patients who had undergone previous ipsilateral hip surgery and in 141 patients who had not. Among patients for whom the surgical approach by Hardinge-Bauer was chosen, there were 146 subjects with HO, and among patients for whom the surgical approach by Watson-Jones was preferred, HO developed in 223 cases. Radiographic signs of HO were found with the following frequencies for each kind of implant: 156 among ceramic-ceramic THR, 75 among Prosthesis with TriboFit^®^ technology, 159 among endoprosthesis.

**Table 1:** Distribution of development of heterotopic ossifications (HO) among patients by rank

Character	Rank	Presence of HO (%)	Absence of HO (%)	*p* values
Age		Mean age = 77.6 years	Mean age = 80.2 years	0.0018
Sex	Male	64.4	35.6	0.1011
	Female	57.7	42.3	
Diagnosis of diseased hip	Coxarthrosis	72.7	27.3	0.0001
	Femoral neck fracture	55.9	44.1	
	Others	82.9	17.1	
Previous HO	Presence	76.2	23.8	0.0260
	Absence	58.8	41.2	
Surgical approach to the hip	Watson-Jones	55.6	44.4	0.0163
	Hardinge-Bauer	65.5	34.5	
Kind of implant	Ceramic-ceramic THR	68.1	31.9	0.0001
	Partial prosthesis	51.3	48.7	
	TriboFit^®^ system	67.0	33.0	
Previous surgery of the ipsilateral hip (only among ceramic-ceramic THR)	Presence	75.0	25.0	0.4896
	Absence	67.5	32.5	

Multivariate analysis of those risk factors that reached statistical significance with the chi-square test was performed. Age (*p* = 0.7483), sex (*p* = 0.3528), diagnosis of diseased hip (*p* = 0.1658) and surgical approach (*p* = 0.0577) did not show to increase HO individually. Statistical significance was reached by the presence of HO after previous hip surgery (*p* = 0.0324) and the kind of implant (*p* = 0.0004). An OR = 2.322 was estimated for those patients with previous ossification. Patients who were treated with TriboFit^®^ technology and ceramic-ceramic THR showed, respectively, an OR = 1.976 and an OR = 1.911 (Table 2). Within the population that showed periarticular HO, each risk factor was assessed for correlation with grading of ossification by Brooker classification. The frequencies obtained are shown in Table 3.

**Table 2:** Odds ratio estimates for those variables that showed to increase the development of periarticular HO individually during multivariate analysis

Effect	Point estimate	95 % Wald confidence limits
TriboFit^®^ vs endoprosthesis	1.976	1.251–3.121
Ceramic-ceramic-coupled THR vs endoprosthesis	1.911	1.326–2.752
Presence of previous HO vs absence of previous HO	2.322	1.073–5.025

*HO* heterotopic ossification

**Table 3:** Distribution of development of heterotopic ossifications (HO) among patients by the grade of HO

Character	Rank	HO grade by Brooker classification (%)				*p* values
		1	2	3	4	
Sex	Male	26.5	28.7	34.5	10.3	0.0478
	Female	39.0	26.8	28.7	5.5	
Diagnosis of diseased hip	Coxarthrosis	40.3	20.8	29.2	9.7	0.3278
	Femoral neck fracture	33.6	28.4	32.1	5.9	
	Others	31.0	34.5	20.7	13.8	
Previous HO	Presence	40.6	38.4	15.6	9.4	0.2830
	Absence	34.1	26.8	32.1	7.0	
Surgical approach to the hip	Watson-Jones	38.1	28.7	26.9	6.3	0.1738
	Hardinge-Bauer	30.8	24.7	37.0	7.5	
Kind of implant	Ceramic-ceramic THR	37.2	25.6	26.9	10.3	0.0093
	Partial prosthesis	35.2	33.3	27.1	4.4	
	TriboFit^®^ system	28.0	18.7	46.7	6.6	

## Discussion

Development of periarticular HO can complicate the postoperative course of healing in those patients who undergo hip replacement surgery. Principal signs and symptoms are local pain and decreased joint mobility, and later, reduced range of motion and ankylosis of the coxofemoral joint may occur. Fortunately, 80 % or more cases of HO run an asymptomatic course. Nevertheless, most studies [1] agree that the radiographic incidence of HO is approximately 53 % of patients undergoing hip replacement without any prophylactic therapy.

**Fig. 4:**
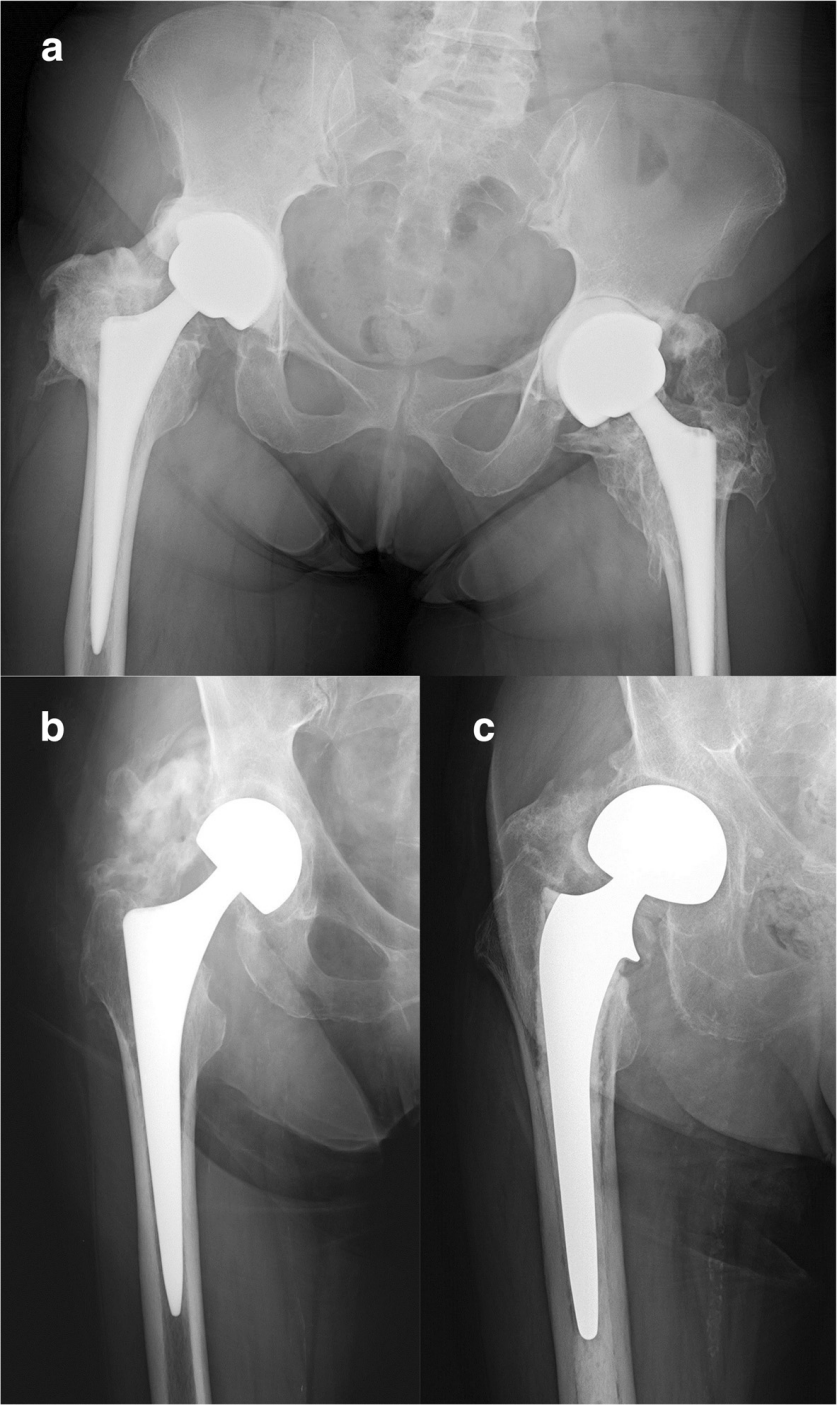
Examples of radiographic high-graded heterotopic ossification for each kind of implant within our cohort. **a** Ceramic-ceramic-coupled total hip replacement. **b** TriboFit^®^. **c** Endoprosthesis

This study reports retrospective data from the radiographic analysis of a cohort of 651 patients who underwent hip replacement surgery with the aim of evaluating the incidence of HO on the basis of different risk factors in the absence of prophylaxis. Discussing our results together with those reported in literature, another purpose is to suggest possible features that make the patients suitable for preventive measures against HO following hip replacement surgery. Of examined patients, 59.9 % (390/651) developed periarticular HO following hip replacement surgery. This result, apparently negative, does not deviate from the mean value reported in similar studies [1] where patients did not undergo any prophylaxis. Instead, an aspect that seems relevant is the high percentage of cases that showed severe or very severe ossification by Brooker classification (38 %), with a variable incidence reported in the literature [7] between 3 and 55 % (Fig. 4). This result could be justified by the choice of surgical approaches that could have contributed negatively. In this study, only two surgical approaches were examined, anterolateral and lateral, both believed liable to cause a higher incidence of HO by several authors [9, 10].

A wide meta-analysis published in the Cochrane Library [14] has proven a reduction of 54–64 % in HO incidence with an adequate prophylactic therapy. If applied to this study, these percentages would decrease our incidence of HO to about 30 %, and it would be a value comparable with those reported by authors [14] who adopted a prophylactic therapy. Thus, our data confirm that the absence of a perioperative prophylaxis seems to be one of the principal causes of a high incidence of HO. The different risk factors implicated in their development are discussed below.

### Age

The mean age of patients who developed HO was 77.6 years, and it was significantly lower (two-sided *p* = 0.0018) when compared to that of patients who did not develop HO (80.2 years). This result refutes some studies [21] which consider older age as a risk factor for HO.

### Sex

Among males, 64.4 % underwent periarticular soft tissue HO versus 57.7 % of females. Even if this trend is in agreement with the literature [1, 5, 22], variance in our study was not statistically relevant (*p* = 0.1011).

### Diagnosis of diseased hip

Statistical significance was reached comparing the percentage of patients who developed HO on the basis of the diagnosis of diseased hip (*p* = 0.0001). Femoral neck fracture predisposes less than coxarthrosis to the development of ectopic bone, probably because operating time is reduced and so are muscular traumatic damage and bleeding. However, the role of some inflammatory pathologies (above all, hypertrophic osteoarthritis) as predictive factors for HO has been well documented in the literature [7]. In fact, inflammation, which is greater in osteoarthritis than in acute events like femoral neck fracture, could justify the difference in the incidences that we found.

### Previous HO

In agreement with the literature [6], patients who developed HO after previous hip surgery showed a higher statistically significant frequency (*p* = 0.0260) of HO than patients with a history of hip surgery but no HO. This aspect can be explained with a subjective predisposition to develop HO.

### Previous surgery of the ipsilateral hip

In contrast to the trend found in the literature [6], among subjects with *ceramic-ceramic THR*, a statistically significant difference was not detected between patients who had undergone prior ipsilateral hip surgery and those who had not (*p* = 0.4896).

### Surgical approach to the hip

The comparison between the two surgical approaches to the hip showed a statistically significant variance on the formation of periarticular HO (*p* = 0.0163). The Hardinge-Bauer surgical approach is a more predisposing factor for HO development than the Watson-Jones surgical approach. However, several studies [9, 10] demonstrate that both surgical approaches predispose the development of HO more than a posterior approach. A recent study [23] found that an anterior minimally invasive approach has some advantages: lower incidence of muscle damage and haematoma, shorter operative and exposure time, less bleeding and immediate rehabilitation. All of these are preventive factors for HO. In particular, prolonged operative time has been associated to an increased development of ectopic bone, such as intraoperative blood loss, even if the latter has not been confirmed by some authors [6, 24]. A minimally invasive anterior approach, where possible, is to be preferred as opposed to lateral or anterolateral approaches, to reduce the formation of HO. The possibility of early rehabilitation given by a minimally invasive technique also seems to be useful in preventing HO [25]. Some pictures taken at the operating table are shown as an example of how important early rehabilitation is (Fig. 3). This patient of our cohort was moved to the emergency room because of her critical condition before (ASA 3) and after the operation and remained motionless for about 1 month in the resuscitation unit. The grade 4 HOs that developed were surgically removed after 1 year.

### Kind of implant

The three different kinds of implants that were objects of study also seemed to have a statistically significant difference in developing ectopic periarticular bone (*p* = 0.0001). While operations carried out with TriboFit^®^ and ceramic-ceramic-coupled THR were complicated by HO in 67 and 68.1 % of cases, respectively, endoprosthesis showed a lower incidence (51.3 %). We did not find comparable data in the literature. Probably, some reasons for this difference are the shorter operative and exposure time and the lesser invasivity (most of all, the loss of the acetabular reaming) that characterizes endoprosthesis when compared to TriboFit^®^ and ceramic-ceramic-coupled THR. The muscle damage, which is more probable when arthroprosthesis is performed, could also play a role in the development of HO. Thus, hip replacement with endoprosthesis reduces the incidence of ectopic ossification; this aspect should be considered when the kind of implant is chosen in borderline cases.

### Multivariate analysis

During the multivariate analysis of those risk factors that reached statistical significance with the chi-square test, variables that showed to individually increase HO incidence were the presence of HO after previous hip surgery (*p* = 0.0324) and the kind of implant (*p* = 0.0004). In particular, an OR = 2.322 was estimated for those patients with previous ossification in comparison to those patients who had undergone prior hip surgery without HO. Patients who were treated with TriboFit^®^ technology and ceramic-ceramic THR showed, respectively, an OR = 1.976 and an OR = 1.911 compared to patients treated with endoprosthesis.

Within the population of subjects that showed periarticular HO, each detected variable was assessed for correlation with the severity of ossification according to Brooker classification. Factors that did not indicate a significant influence on grading of ossification were diagnosis of diseased hip (*p* = 0.3278), surgical approach to the hip (*p* = 0.1738) and HO due to previous hip surgery (*p* = 0.2830).

### Development of HO following Brooker classification

On the other hand, association between the severity of HO and the following factors turned out to be statistically significant:

1. Kind of implant (*p* = 0.0093). In particular, with patients who underwent ceramic-ceramic-coupled THR and TriboFit^®^, respectively, 37.2 and 53.3 % of cases developed ossification of grades 3 and 4, versus 31.4 % of patients whose implant was an endoprosthesis.
2. Gender (*p* = 0.0478). Male patients showed a percentage of 44.8 % of severe or very severe ossification, versus 34.2 % of female patients, in agreement with literature data [1, 5, 22].

Some data presented in this study may encourage improvement of the prophylactic drugs against formation of periarticular HO and early rehabilitation. However, our study shows some weaknesses, which should be pointed out. First of all, we did not consider the clinical counterpart of the HOs found. Usually, radiographic HO is not related to clinical course and most HOs occur asymptomatically; they may, however, involve clinical impairment, such as decreased hip range of motion and function and pain [26–28]. Hence, we strongly believe that further research is necessary to establish a relationship between the radiographic grade of HO and its clinical aspect. Another apparent point of weakness is that we considered the presence in our computerized database of a minimum follow-up control at 6 months from hip surgery as an inclusion criterion (only nine patients with a minimum follow-up of 6 months, seven patients with an 8-month follow-up and six with a 10-month follow-up). Considering this period reasonable to observe or predict the development of HO, we could have slightly underestimated the number of patients which developed HO and, in particular, the number of severe and very severe HOs (grades 3 and 4 by Brooker classification). However, HO is usually evident from radiographs by 6 weeks after surgery. Hence, the ossification matures throughout the first 6 months and then generally does not develop further thereafter [13, 26, 27].

Finally, the fact that our institute is a trauma clinic could have introduced some difficulties of interpretation. In particular, 80 % of the population studied suffered from femoral neck fracture and only 15 % from coxarthrosis. A bias towards endoprosthesis in fracture and THR in hip osteoarthrosis is plausible. However, we used logistic regression as our statistical modelling method to control confounding variables as much as possible. According to our knowledge, in the literature, there are no comparable data about the relationship between HO and the kind of implants considered in this study. This variable was shown to affect the development of HO in our research, and further studies are needed to confirm our results. Moreover, the presence of a higher incidence of HO in African-American patients following acetabular fracture surgery [12] and in the Japanese population because of multiple injuries, nerve injuries and ossificans diseases [2–4] should be investigated further. In addition, our data should be compared with other ethnic groups as our study was conducted only with a cohort of Caucasian subjects.

## Conclusions

The development of HO following hip replacement surgery is affected by endogenous and exogenous factors. Our radiographic study confirms, in agreement with the literature, some risk factors, such as previous HO of soft tissue around the hip, kind of implant, surgical approach to the hip, diagnosis of coxarthrosis versus femoral neck fracture and male gender. On the other hand, there are factors in contrast with some of the authors’ outcomes, such as older age. In particular, Hardinge-Bauer and Watson-Jones surgical approaches, characterized by a wide exposure of the coxofemoral joint, and ceramic-ceramic THR and TriboFit^®^ significantly favour the formation of HO. Thus, we think that orthopaedic surgeons should prefer alternative surgical approaches and minimally invasive implants. Furthermore, our results and literature data suggest that a prophylactic treatment could be a choice to drastically decrease the incidence of HO after hip replacement surgery. We believe it is necessary to carry out a controlled and randomized study as a valid aid for the choice of the most appropriate therapeutic option according to different categories of patients.

## Abbreviations

HO: heterotopic ossification
NSAIDs: nonsteroidal anti-inflammatory drugs
THR: total hip replacement
